# Comparing venous wall effects using the empty vein ablation technique with VELEX catheter, endovenous laser ablation and foam sclerotherapy in an animal model

**DOI:** 10.1016/j.jvsv.2025.102251

**Published:** 2025-04-23

**Authors:** Mario Salerno, Daniele Bissacco, Yung-Wei Chi, Sriram Narayanan, Alessandro Addis, Fabio Martelli, Germana Zaccagnini, Teresa Lucia Aloi, Giovanni Nano, Sergio Gianesini, Paolo Righini

**Affiliations:** a Unit of Angiology, Department of Medicine and Cardiopulmonary Rehabilitation, Istituti Clinici Scientifici Maugeri IRCCS, Institute of Tradate, Varese, Italy; b I-VASC S.r.l, Milan, Italy; c Department of Clinical Sciences and Community Health, University of Milan, Milan, Italy; d Vascular Center, University of California, Davis, CA; e The Harley Street Heart and Vascular Center, Singapore; f CRABCC, Biotechnology Research Center for Cardiothoracic Applications, Rivolta D’Adda, Cremona, Italy; g Molecular Cardiology Laboratory, IRCCS Policlinico San Donato, San Donato Milanese, Milan, Italy; h Cardio-Angiology Unit of Montescano and Pavia Institute, Istituti Clinici Scientifici Maugeri Istituto di Ricerca e Cura a Carattere Scientifico (IRCCS), Pavia, Italy; i Department of Biomedical Sciences for Health, University of Milan, Milan, Italy; j Operative Unit of Vascular Surgery, IRCCS Policlinico San Donato, San Donato Milanese, Milan, Italy; k Department of Translational Medicine, University of Ferrara, Ferrara, Italy; l Department of Surgery, Uniformed Services University of Health Sciences, Bethesda, MD

**Keywords:** Empty vein ablation, Varicose veins, Sclerotherapy, Chronic venous disease

## Abstract

**Objective:**

To describe residual intima and the average media thickness persisted after the empty vein ablation (EVA) technique, endovenous laser ablation (EVLA), and foam sclerotherapy (FS) in a sheep in vivo model.

**Methods:**

Six iliofemoral and two jugular sheep vein axes were treated as follows: four with EVA (using polidocanol [POL] 0.5% or 1% with 1 or 3 minutes as contact time), two with FS (FS-1 and FS during Valsalva maneuver [FS-Val], POL1% for 10 minutes), and two with EVLA (1470 nm radial, 80 J/cm^2^).

**Results:**

The average percentage of residual intima layer was 2% (interquartile range [IQR]: 1%-4%) for EVA-POL0.5%-1 minute, 1% (IQR: 0%-3.5%) for EVA-POL0.5%-3 minutes, 2% (IQR: 0%-4%) for EVA-POL1%-1 minute, 0 for EVA-POL1%-3 minutes, 13% (IQR: 13%-15.7%) for FS, 1% (IQR: 0%-3%) for FS-Val, and 1% (IQR: 0%-6%) for EVLA. The average percentage of residual media thickness was 13% (IQR: 8%-15%) for EVA-POL0.5%-1 minute, 6% (IQR: 4%-9%) for EVA-POL0.5%-3 minutes, 13% (IQR: 10%-27%) for EVA-POL1%-1 minute, 6% (IQR: 5%-12%) for EVA-POL1%-3 minutes, 51% (IQR: 40%-62%) for FS, 29% (IQR: 23%-35%) for FS-Val, and 62% (IQR: 41%-75%) for EVLA.

**Conclusions:**

EVA demonstrated better results in vein wall damage compared with EVLA and FS, both in intima and media layers.

**Clinical Relevance:**

This study provides crucial insights into the effectiveness of different vein treatment techniques, particularly the empty vein ablation method, in minimizing residual intima and media thickness. By evaluating these outcomes in a sheep model, it highlights how empty vein ablation may lead to more vein wall damage compared with endovenous laser ablation and foam sclerotherapy. For clinicians, understanding the comparative efficacy of these treatments is vital for optimizing patient care in managing venous diseases. As the field evolves, these findings could influence clinical decision-making, encouraging the adoption of techniques that promote better long-term outcomes for patients.

**Article Highlights**

• **Type of Research:** Animal experimental study
• **Key Findings:** The study assessed residual post-treatment circumferential residual intima and the average media thickness in sheep veins after empty vein ablation (EVA), endovenous laser ablation, and foam sclerotherapy. Results showed that EVA, particularly with 1% polidocanol and longer contact times, resulted in the best wall damage in both intima and media.
• **Take Home Message:** The EVA technique demonstrated superior efficacy in venous wall damage, combining and maximizing chemical (sclerosant) and physical (balloon denudation) effects on both intima and media layers, and so providing better results in target points for effective and long-lasting results after sclerotherapy.

Chronic venous disease is a widespread condition that affects the lower limbs and has a substantial impact on both patient well-being and national health care budgets.^1^^,^^2^ The preferred method for treating chronic venous disease is endovenous thermal ablation, which uses laser (EVLA) or radiofrequency technology.^1^

Although alternative nonthermal, nontumescent techniques have been proposed to alleviate the discomfort associated with tumescent anesthesia and to minimize the treatment impact, further evidence is needed to determine whether these techniques can achieve occlusion rates comparable to endovenous thermal methods. Indeed, in terms of occlusion rate, sclerotherapy has been found to be less effective than thermal ablation techniques.^3^ Despite foam sclerotherapy (FS) has been proven safe and better than the liquid form alone injection,^4^ a significant limitation remains the inadequate damage to the venous wall. This results in insufficient activation of thrombogenic processes, which are necessary for effectively occluding the target vein and achieving lasting results by fibrotic thrombus transformation.

The empty vein ablation (EVA) technique has been proposed as a method that may overcome several limitations associated with sclerotherapy, maximizing its effect.

Previous studies have suggested that EVA could address issues such as suboptimal penetration of the sclerosing agent (SA) into the vein wall depth, inactivation of the SA by plasma proteins or dilution/flushing by the blood stream, heterogeneous SA concentration and contact along the vein wall circumference, and insufficient contact time between the SA and the inner vein wall.

Specifically, an ex vivo study has shown that EVA resulted in a significantly lower percentage of intact circumferential residual intima compared with FS, causing more uniform damage throughout the treated vein segment, whereas FS had less destructive effects distally from the injection site.^5^ A second in vivo animal study aimed to analyze the effects of EVA on the intima and media layers of the vessel wall using different sclerosing agent concentrations (SLAC) and contact times (ctSA/VW). The results showed that EVA was effective in destroying the venous wall even with very low SLAC (0.5% polidocanol [POL]) and short ctSA/VW (3 minutes) in both intima and media layers, even in quite large caliber veins (8-13 mm).^6^

Once the success of EVA has been proven, it was necessary to provide useful data on the comparison with other endovenous techniques, in terms of wall disruptive effects, starting from animal models. Moreover, although previous studies focused on using EVA to treat single short segments, it was essential to evaluate its effectiveness on longer segments. This was accomplished through sequential pullback, a process that will be explained in detail later.

The aim of this paper is to evaluate and compare, in terms of the percentage of residual intima and media layers after the procedure, EVA—using different SLAC and ctSA/VW—with FS and EVLA, in long vein segments.

## Methods

### Device description

**Fig 1:**
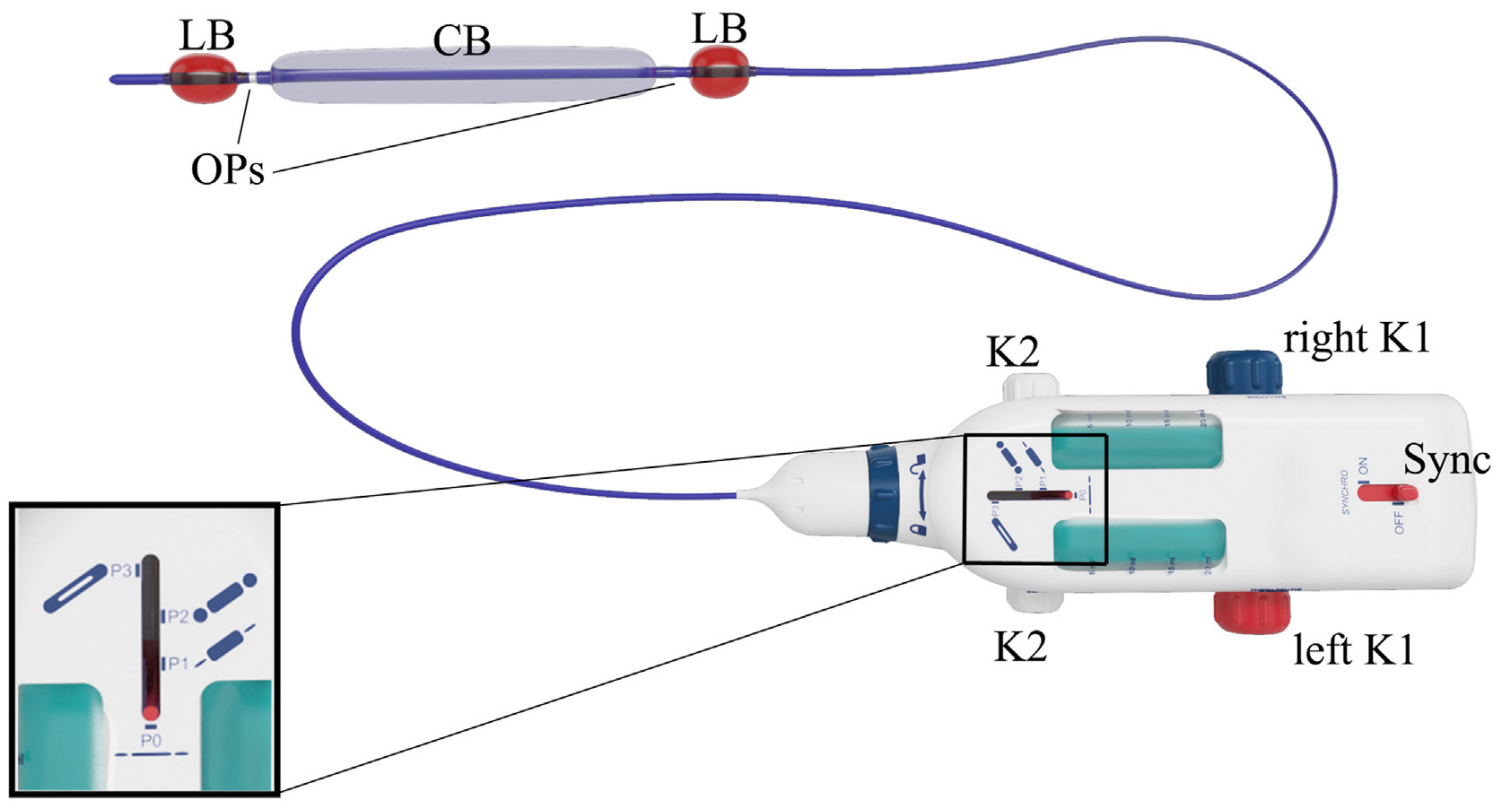
Empty vein ablation device. The zoomed box shows details of the selector for balloon inflation/deflation and sclerosing agent injection. *CB*, Central balloon; *K1*, posterior routable knobs; *K2*, anterior routable knobs; *LB*, lateral balloon; *OP*, opening for sclerosing agent release; *Sync*, synchronizer.

In brief, the EVA procedure uses a custom-designed device comprising a three-way, three-balloon catheter and a handpiece (Fig 1). The 850-mm-long, 2-mm-diameter catheter features a central cylindrical balloon (CB) and two lateral spherical balloons (LBs). The CB displaces blood laterally, whereas the LBs isolate the empty vein segment once the CB is deflated. The catheter has three lines for CB inflation, LB inflation with saline, and SA delivery. The handpiece manages the inflation/deflation of the balloons and the SA infusion, with a synchronizer that enables the syringes to work in reverse synchrony.

**Fig 2:**
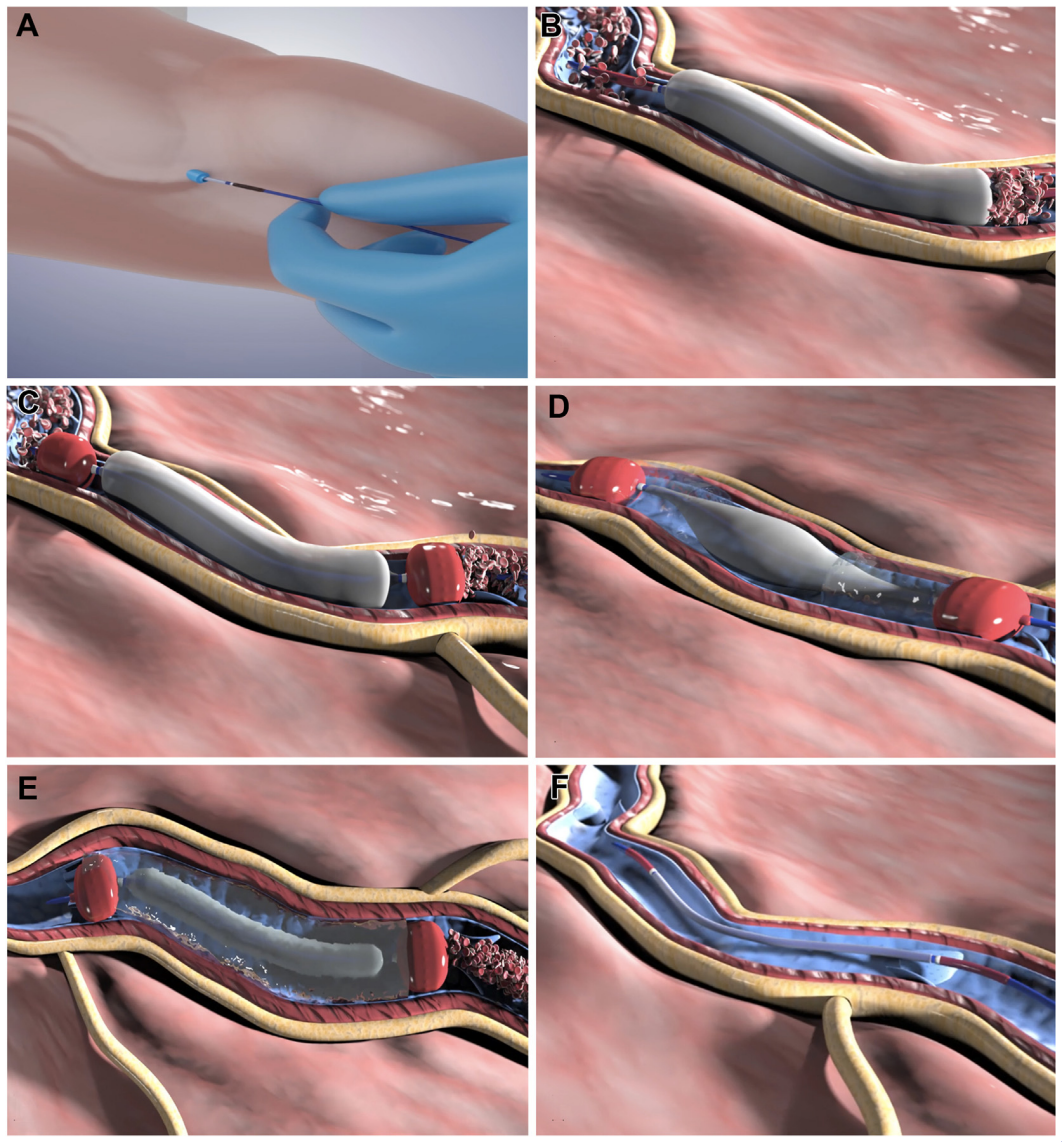
Empty vein ablation (EVA) technique summary steps. **A,** The catheter is inserted in the vessel to be treated by percutaneous access, through a 7F introducer sheath; three balloons are not inflated. **B,** Central balloon is inflated first, causing the blood to move and leave that vein segment. **C,** The two lateral balloons are subsequently inflated to isolate the segment of vein previously emptied from blood. In this way an empty and isolated vein segment is obtained. **D,** Medication injection is started, through the two openings, into the “central chamber” interposed between the two lateral balloons and through a partial deflation of the middle balloon. **E,** At the end of the vein treatment, the central balloon is inflated again, and simultaneously, the medication is recaptured to empty the vessel from it. **F,** At the end of the procedure, the three balloons are deflated simultaneously in order to return to the initial configuration, and the catheter is withdrawn.

The EVA procedure (Fig 2) involves filling the handpiece syringes with saline or SA, connecting the catheter to prepare the system. Venous access is obtained using the Seldinger technique under Duplex ultrasound guidance. Once inserted into the vein at the target level, the CB is inflated to empty the target vein segment, followed by inflation of the LBs in contact with the vein wall. SA is injected into the central empty space between the LBs while simultaneously deflating the CB. The desired quantity of SA is infused and allowed to dwell for the defined therapeutic time. For multiple vein segments, the device can be pulled back along the vein while keeping the LBs inflated to reuse the same SA cylinder. Indeed, after completing the first segment, the catheter with the inflated LBs and the SA cylinder positioned between them is translated by a length identical to that of the chamber (13 cm) to allow treatment of the segment adjacent to the first. The device is held in position for a therapeutic time (1 or 3 minutes). The translation operation is then repeated if necessary to treat additional venous segments.

Unlike previous studies,^5^^,^^6^ as mentioned, in these experiments, multiple segments were treated with EVA by pulling back the device along the target vein, according to the instruction for use.

### In vivo model

The experiments followed the updated guidelines for reporting animal research (ARRIVE) guidelines.^7^ The experimental procedures performed in this study were conducted in accordance with Italian law D.Lgs 26/14 and European Union Directive 2010/63/UE on the protection of animals used for scientific purposes. The study received ethical approval from the Italian Ministry of Health (authorization number 1025/2020-PR of October 27, 2020). The animals were obtained with permission from the Biotechnology Research Center for Cardiothoracic Applications (CRABCC, Rivolta d’Adda, Cremona, Italy). Adult female sheep *Bergamasca* breed (average weight 75-80 kg) have been used to test the efficacy of EVA, FS, and EVLA in destroying the intima and media vein wall. All procedures were performed under general anesthesia by a skilled vascular surgeon in endovenous techniques.

### EVA technique

EVA was used to target four iliofemoral venous axes in two sheep. The treated veins had an average length of 35 cm and a diameter of 12 mm. Various SAs were combined and tested, including POL at SLAC of 0.5% and 1%, as well as different ctSA/VW (1 or 3 minutes).

As mentioned, the treatment technique used a stepwise withdrawal of the catheter, with the SA being injected between the two LBs along the iliofemoral axis (in two or three steps) to ensure comprehensive treatment of the entire vein segment.

### Foam sclerotherapy

In addition, one adult sheep underwent FS using the right and left iliofemoral venous axes, also approximately 35 cm in length and 12 mm in diameter. The FS was prepared according to the Tessari method, using 2 cc of 1% POL and 8 cc of ambient air. After isolating the femoral veins, a 6F catheter was inserted, and 10 cc of FS agents were injected into the right iliac axis, allowing the medication to circulate freely within the bloodstream. In the left iliac axis, 10 cc of FS agents (Tessari method) were injected, and the Valsalva maneuver was simultaneously performed for 3 minutes to increase the contact time between the SA and vein wall by decreasing venous flow (FS-Val). In particular, the tests were conducted with mechanical ventilation in place. During the Valsalva maneuver, the ventilator was briefly paused to allow forced insufflation, which extended the expiration phase.

Recommendations from recent guidelines were followed.^8^^,^^9^

### EVLA method

Finally, a second adult sheep was treated at the level of the jugular veins (15 cm in length, 12 mm in diameter) using a 1470-nm radial EVLA with a 600-nm optical fiber, delivering 80 J/cm (pullback of 1 mm/s at 8 W) under previous tumescence anesthesia. Recommendations from recent guidelines were followed.^3^^,^^10^

The treated segments were explanted approximately 1 hour after the end of the procedures and were preserved in 10% formalin. For each vein segment, proximal, middle, and distal samples were analyzed. We defined “proximal” as the segment closer to the device access site.

### Preparation of samples for histological analysis

Regarding the EVA treatment, four segments were provided for each venous section: proximal, mid-proximal, mid-distal, and distal. For histological analysis, these segments were further divided into subsegments of a length suitable for inclusion in paraffin blocks, with 3 to 4 subsegments analyzed per segment (N = 3/4 for each segment). The paraffin blocks were selected for analysis to ensure that the sampling represented various areas of the segment (not all adjacent). Samples eventually unsuitable for the analysis, due to technical issues, were not analyzed. The results from the mid-proximal and mid-distal segments were combined under the label “middle” to standardize them with the others in the graphs.

For the foam, Valsalva foam, and laser treatments, three segments were provided for each venous section: proximal, middle, and distal. Like the EVA treatment, these segments were divided into subsegments appropriate for paraffin inclusion, with 3 to 4 subsegments analyzed for a total of 10 per segment (N = 3/4 for each segment). The sampling was designed to represent different areas of the segment. The evaluation was conducted to randomly sample the entire venous section effectively.

### Histological analysis

Formalin-fixed, paraffin-embedded consecutive sections (3 μm thickness) were dewaxed, hydrated through a graded decreasing alcohol series, and stained for histological analysis. Hematoxylin/eosin staining was performed using a standard protocol (Mayer’s Hematoxylin, #05-06002/L; Eosin #05-10002/L; Bio-Optica). Masson’s trichrome staining with aniline blue (#04-010802 Bio-Optica) was performed following the manufacturer instructions. Slides were acquired with an Aperio AT2 digital scanner at a magnification of ×200 (Leica Biosystems). Morphology assessment focused on the determination of endothelium percentage persistence after treatment, as assessed on hematoxylin-eosin-stained sections by a dedicated imaging platform (Aperio ImageScope; Leica Biosystems) after recommendations from Ikponmwosa et al^11^ and the histological evaluations proposed by Erkin et al.^12^ The same imaging platform was used to evaluate the effect on media, using Masson’s trichrome-stained slide.

### Intima and media measurements

The measurements of the residual endothelium and lumen perimeter, as well as the measurements of the media thickness, were performed manually on the basis of morphological criteria.

**Fig 3:**
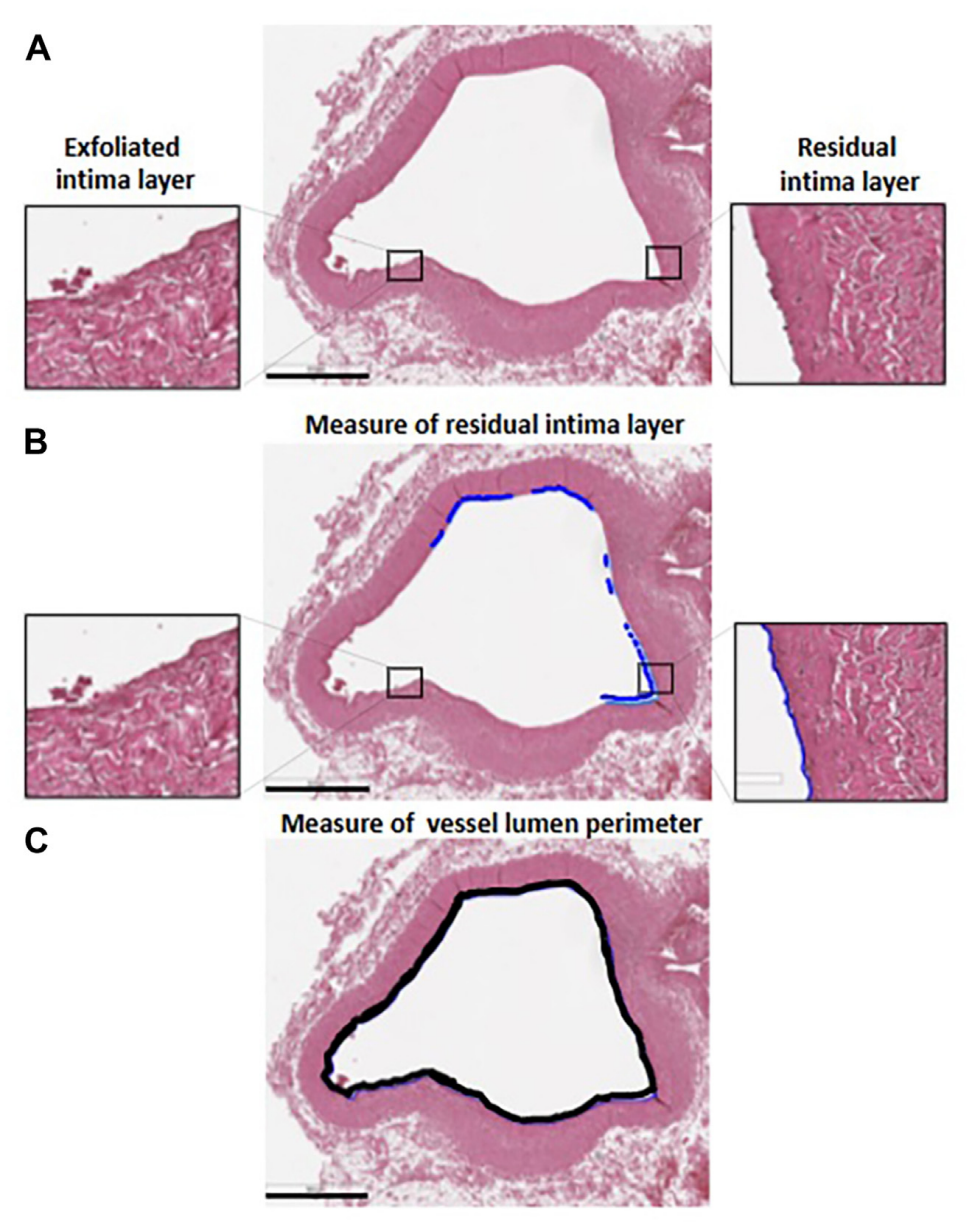
**A,** Representative pictures of H/E-stained sections of the femoral vein. On the left, the inset shows the morphology of a stretch of the exfoliated intima layer at high magnification. On the right, the inset shows the morphology of a stretch of the residual intima layer at high magnification. **B,** The stretch of the residual intima layer was measured by Aperio Image Scope software. **C,** Measure of the vessel lumen perimeter, carried out by Aperio Image Scope software. Calibration bar 800 μm.

For the intima layer (Fig 3), segments of residual (preserved) endothelium were measured and summed to determine the total length of preserved endothelium. This total length was divided by the lumen perimeter and expressed as a percentage, representing the proportion of preserved endothelium.

**Fig 4:**
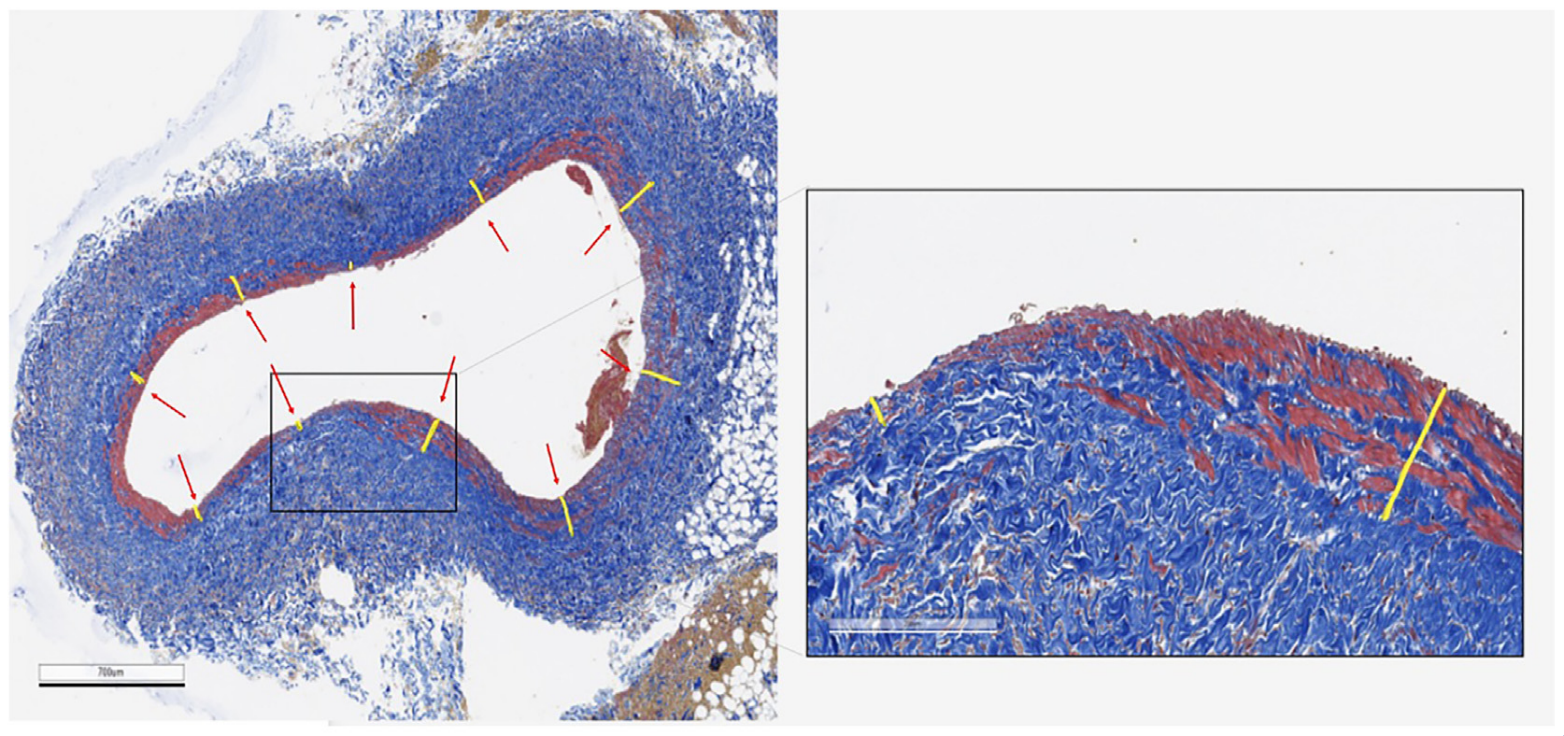
Representative images of Masson trichrome-stained femoral vein sections. Ten measurements of the media thickness were taken for each vessel at equivalent distances along the lumen. If the tunica media was completely absent at the measurement point, the thickness measurement was assigned to a value of zero. Calibration bar 700 μm. The inset shows the magnification of the indicated area; calibration bar 200 μm.

For the evaluation of the media thickness, reference values were obtained measuring the thickness of the media on treated vessels along the segments of preserved endothelium (not damaged by the treatment). The average of these values was used as control. In assessing the thickness of the media layer (Fig 4), 10 measurements of media thickness were taken on each vessel at evenly spaced intervals to comprehensively cover the entire perimeter. In regions where the media layer was absent, a value of zero was recorded. This approach was adopted because of the differing morphological characteristics between the femoral and jugular veins, making percentage representation a clearer means of comparative analysis.

The primary outcomes were the circumferential residual intima and the average media thickness—both expressed as percentages— persisted after EVA (POL0.5%-1 minute, POL0.5%-3 minutes, POL1%-1 minute, and POL1%-3 minutes), FS, FS-Val, and EVLA.

### Statistical analysis and raw data availability

Data analysis was performed using GraphPad Prism v.4.03 software (GraphPad Software Inc). Continuous variables were expressed by median with interquartile range (IQR) or mean ± standard deviation in accordance with their distribution, whereas nominal variables as number (%).

A D’Agostino-Pearson test for normality was first performed, revealing that the data were not normally distributed across all groups. Given this nonparametric nature of the data, a Dunn’s Kruskal-Wallis test with multiple comparisons was used to compare the EVA, EVLA, FS, and FS-Val groups. A *P* value of <.05 was considered statistically significant.

Raw data were uploaded to a data repository before submission and uploaded during revision (10.5281/zenodo.15082844).

## Results

All procedures were successfully performed. No perioperative complications occurred. In total, 85 and 84 samples were used for the intima and media analyses. Among these, 1 sample for intima and 1 sample for media did not retrieve acceptable tissue, so they were not used.

**Fig 5:**
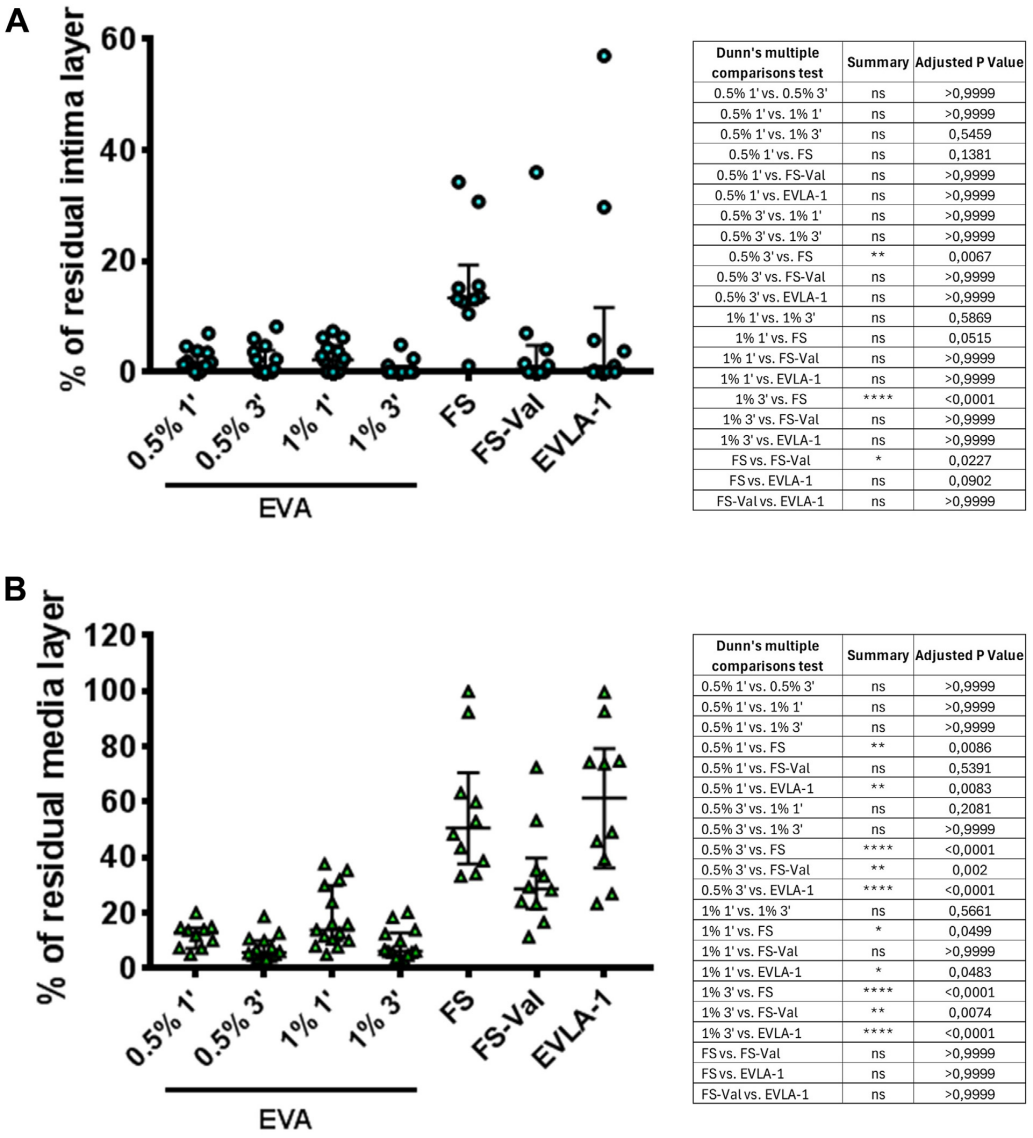
Median with interquartile range (IQR) of the average percentage of residual intima **(A)** or residual media thickness **(B)** layer after empty vein ablation (*EVA*)—used with polidocanol (POL) 1% or 3% for 1 or 3 minutes—foam sclerotherapy (*FS*)—with or without Valsalva (*Val*) maneuver—and endovenous laser ablation (*EVLA*). ∗*P* < .05, ∗∗*P* < .01, ∗∗∗*P* < .0001. *ns*, Not significant.

The average percentage of residual intima layer (Fig 5, *A*) was 2% (IQR: 1%-4%) for EVA-POL0.5%-1 minute, 1% (IQR: 0%-3.5%) for EVA-POL0.5%-3 minutes, 2% (IQR: 0%-4%) for EVA-POL1%-1 minute, 0 for EVA-POL1%-3 minutes, 13% (IQR: 13%-15.7%) for FS, 1% (IQR: 0%-3%) for FS-Val, and 1% (IQR: 0%-6%) for EVLA.

The average percentage of residual media thickness (Fig 5, *B*) was 13% (IQR: 8%-15%) for EVA-POL0.5%-1 minute, 6% (IQR: 4%-9%) for EVA-POL0.5%-3 minutes, 13% (IQR: 10%-27%) for EVA-POL1%-1 minute, 6% (IQR: 5%-12%) for EVA-POL1%-3 minutes, 51% (IQR: 40%-62%) for FS, 29% (IQR: 23%-35%) for FS-Val, and 62% (IQR: 41%-75%) for EVLA.

## Discussion

Historically, the primary goal of all endovenous techniques has been to damage the venous wall. This damage is crucial, as it leads to the destruction of the endothelium and the subsequent exposure of the underlying subendothelial collagen, which activates the intrinsic pathway of the coagulation cascade. This process results in the formation of a “sclerothrombus,” causing thrombotic occlusion of the lumen and ultimately leading to vein fibrosis and the cessation of venous reflux. The necessity of achieving transmural death of the vein wall for effective long-term ablation has been consistently emphasized by multiple authors over the years.^11^^,^^13^, ^14^, ^15^ Interestingly, this transmural damage explains why sclerotherapy tends to be more successful in smaller veins with thinner walls compared with larger veins that have thicker walls.

These preliminary findings offer valuable insights by directly comparing the destructive effects of various ablative techniques on venous walls, including the depth and extent of damage. Additionally, they highlight the performance of the EVA technique, demonstrating its applicability in a manner similar to human settings.

Starting from the tunica intima, our previous article on the EVA technique in an in vivo animal model argued that EVA using POL 0.5% (3 minutes), 0.5% (5 minutes), 1% (3 minutes), and 1% (5 minutes) resulted in a residual intima of 21.3% ± 4.9%, 18.2% ± 7.4%, 15.7% ± 2.4%, and 8.9% ± 2.0%, respectively.^6^ These results have not only been reaffirmed by this new analysis but also enhanced, achieving nearly 0 with POL1%-3 minutes. This advancement can be attributed to the technique used for EVA. Unlike previous studies that have used EVA in a static manner, this research involved the device pullback—similar to the approach taken with thermal ablation with radiofrequency or EVLA catheters—before applying a new segment treatment. This withdrawal might have caused additional damage to the intima through the passage of the LBs throughout the treated vein segment, potentially exacerbating wall injury.

The concept of “balloon denudation” (BD) serves as a widely recognized model for investigating vascular responses to injury, particularly in arterial contexts.^16^ It induces smooth muscle cell proliferation and the development of intimal hyperplasia. Furthermore, balloon-induced injury disrupts endothelial-dependent vessel relaxation and can trigger vessel spasms.^17^ In venous applications, this technique has been recommended to enhance the efficacy of sclerotherapy, leading to an approximate 10% and 6% increase in endothelial cell loss, followed by a 5-minute exposure to 1% and 3% sodium tetradecyl sulphate, respectively.^18^ Conversely, in this paper, BD was used after (and not before) sclerotherapy, increasing its efficacy.

Intima remains a primary target for achieving efficient results in endovenous techniques. With the use of EVA, it was effectively eliminated (all IQR below 5%), regardless of the concentration of POL or the duration of contact between the SA and the vein wall. Among the techniques evaluated, FS-Val emerged as the second most effective method. The Valsalva maneuver during sclerotherapy enhances the contact time between the SA and the venous wall (by blocking the blood flow), a critical factor influencing the success of the procedure. However, this maneuver may pose risks in humans, as it creates a suction force at the conclusion of the Valsalva that can draw the slower flow of the great saphenous vein toward the faster flow in the deep veins, potentially displacing the SA all at once, with potential systemic complications.^19^ The wall effects of EVLA and FS techniques have been extensively studied, primarily through qualitative analyses that describe the types of damage but rarely quantify them.^20^^,^^21^ Regardless of the technique used, the superficial damage remains vigorous, with low residual intima in all techniques but FS.

A more aggressive approach to the intima leads to increased exposure of the tunica media. The tunica media is a crucial target for achieving lasting results, as it plays a key role in thrombus formation and the subsequent endovenous fibrosis that occurs postoperatively.^22^

EVA proved effective in damaging the tunica media, with residual average wall thickness remaining quite low, regardless of the sclerosant agent concentration or contact time. Notably, contact time appears to be a more critical factor for effectiveness than concentration. This result is also observed, albeit to a lesser degree, in the intimal area, supporting findings from previous studies.^21^^,^^23^ While FS-Val showed the second-best results, other techniques resulted in a residual average media thickness exceeding 50%.

To summarize, EVA preliminarily demonstrates the ability to maximize damage to both the endothelium and the muscular layer of a vein, regardless of its length (starting from 13 cm and its multiples). This is achieved through the innovative capability of performing a pullback of pure sclerosing liquid at a consistent concentration for the first time. The traction mechanism of EVA enhances its effectiveness by leveraging the mechanical abrasion effect of the balloon on the chemically treated tissue (BD). Moreover, data comparing the extent of injury to both the endothelium and muscular layer show that EVA outperforms results from previous static tests conducted on a single 13 cm segment.^5^^,^^6^ Finally, the damaging effects observed with EVA seems to surpass those achieved with the two established gold standard treatments: thermal alone (EVLA) and chemical alone (FS) methods.

The abovementioned device mechanism and results could be considered a really new technology in providing venous ablation. Indeed, EVA combines and enhances sclerosing effects and mechanical abrasion on both intima and media, increasing the effectiveness and durability of the vein wall damage. Despite this, several studies continue to show a high rate of obliteration in the medium to long term for thermal techniques.^24^ These studies indicate that damage persists even after the acute treatment phase, leading to wall remodeling and venous obliteration that can occur even 48 to 72 hours later.^25^ In other words, a less detrimental impact on the venous wall for EVLA was seen if compared with EVA, but this does not necessarily imply that clinical outcomes will be worse. However, if EVA causes greater damage than EVLA in the acute phase, the long-term results should be at least comparable to this technique.

### Limitations

**Fig 6:**
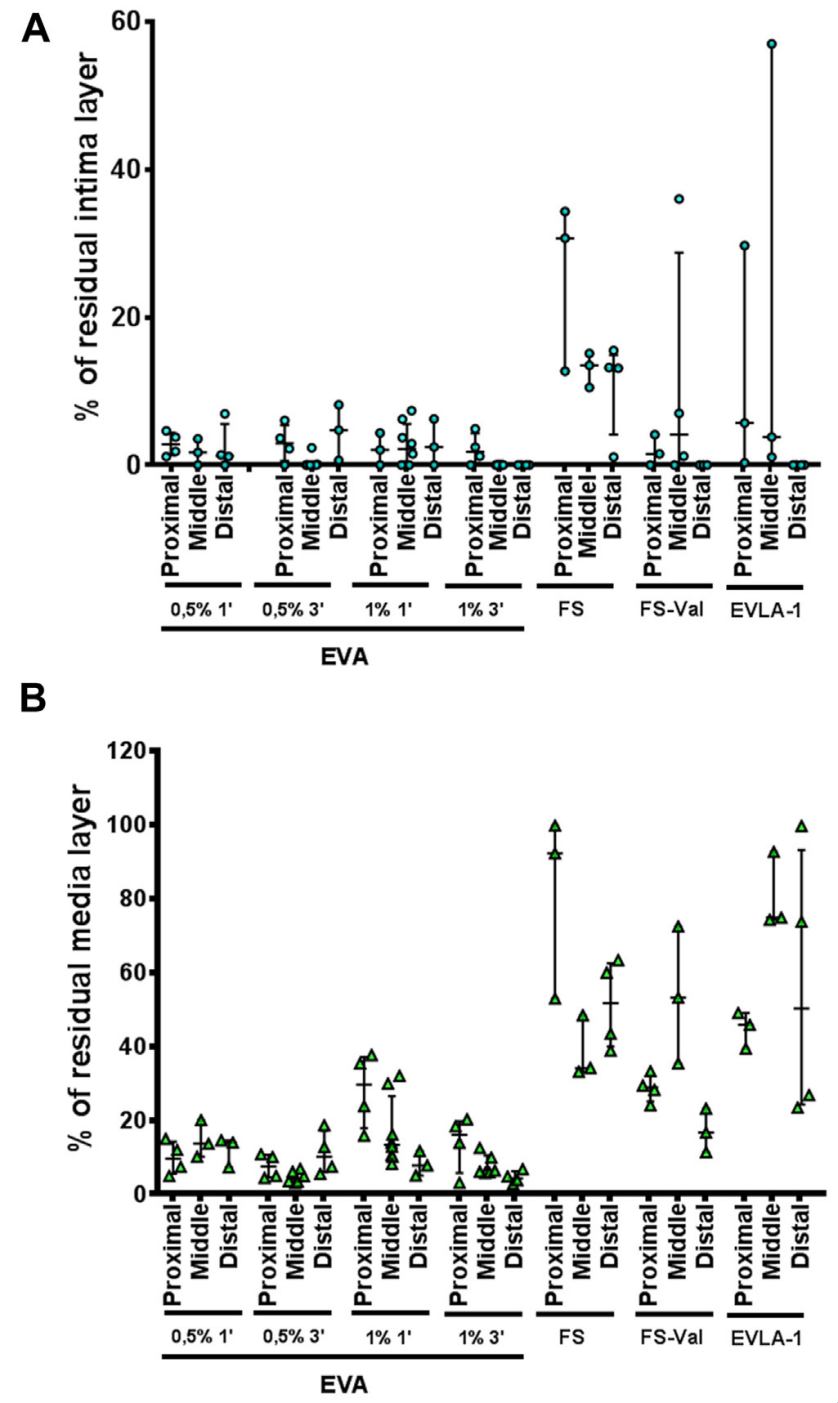
Evaluation of residual intima **(A)** or residual media thickness **(B)** along the vessel, dividing each treated segment into proximal, medial, and distal portion after empty vein ablation (*EVA*)—used with polidocanol (POL) 1% or 3% for 1 or 3 minutes—foam sclerotherapy (*FS*)—with or without Valsalva (*Val*) maneuver—and endovenous laser ablation (*EVLA*). Values are represented as median with interquartile range (IQR).

As a pivotal experience, results could be considered preliminary and need to be confirmed by further analysis, assessing variability not only between techniques but also in multiple attempts with the same technique, particularly regarding the net advantage using the BD maneuver. Moreover, when the percentage of residual media is declared, it is not described how much damage goes into the wall, giving more affected areas than less affected areas, both along the treated vein and in the same analyzed segment. Despite this limitation, the division into proximal, medium, and distal samples provides us with further information on the homogeneity of the damage (Fig 6), although without clinical impactful results.

Finally, a complete comparison between a healthy sheep deep vein and an incompetent human saphenous trunk, which is the target of the EVA technique, is inherently challenging. This presents a limitation in our study. However, the primary objective of this pilot study was to assess the transmural efficacy of EVA and the BD technique in relation to established methods. Future studies involving human subjects will undoubtedly yield valuable insights, particularly regarding the clinical—and not only histological—efficacy of the significant damage inflicted on the venous wall.

## Conclusions

The findings indicate that EVA consistently achieved minimal residual intimal, with percentages significantly lower than those observed with FS and EVLA. In addition, EVA demonstrated a more effective impact on the media layer, further establishing its potential as a preferred method for venous treatment. These results suggest that EVA could be a valuable technique for enhancing the outcomes of venous interventions, warranting further investigation in larger clinical studies to confirm its benefits in human applications.

## Author contributions

Conception and design: MS, DB, YC, SN, TA, GN, SG, PR

Analysis and interpretation: MS, DB, YC, SN, AA, FM, GZ, TA, GN, SG, PR

Data collection: MS, AA

Writing the article: MS, DB, AA, FM, GZ

Critical revision of the article: MS, DB, YC, SN, AA, FM, GZ, TA, GN, SG, PR

Final approval of the article: MS, DB, YC, SN, AA, FM, GZ, TA, GN, SG, PR

Statistical analysis: DB, FM, GZ

Obtained funding: MS

Overall responsibility: MS

## Funding

Members of I-VASC S.r.l. were involved in the development of the manuscript. However, statistical and outcomes analyses were provided by independent research authors (F.M. and G.Z.). Publication writing and statistical analysis have been funded by I-VASC S.r.l. Other authors report no involvement in the research by the sponsor that could have influenced the outcome of this work. F.M. is supported by the 10.13039/501100003196Italian Ministry of Health, Ricerca Corrente 2024 1.07.128, RF-2019-12368521, and POS-T4 CAL.HUB.RIA T4-AN-09; and by the Next Generation EU-NRRP M6C2 Inv. 2.1 PNRRMAD 2022-12375790, PNRR-MCNT2-2023-12377983, and EU PNRR/2022/C9/MCID/I8, 10.13039/501100000780European Commission.

## Disclosures

M.S. is inventor of empty vein ablation technology and the founder, CMO, and board member of I-VASC S.r.l., which is developing the VELEX device for commercial uses. The remaining authors report no conflicts.
